# A semi-parametric approach to estimate risk functions associated with multi-dimensional exposure profiles: application to smoking and lung cancer

**DOI:** 10.1186/1471-2288-13-129

**Published:** 2013-10-23

**Authors:** David I Hastie, Silvia Liverani, Lamiae Azizi, Sylvia Richardson, Isabelle Stücker

**Affiliations:** 1 Imperial College, London, UK; 2 MRC Biostatistics Unit, Cambridge, UK; 3 Unite d’Epidemiologie Animale, INRA-Theix, Saint-Genès-Champanelle, France; 4 INSERM, Centre for Research in Epidemiology and Population Health (CESP), U1018, Environmental Epidemiology of Cancer Team, Paris, France; 5 Université Paris-Sud, UMRS 1018, F-94807, Villejuif, France

**Keywords:** Smoking, Lung cancer, Bayesian clustering, Case control study, Intensity, Duration, Pack-years

## Abstract

**Background:**

A common characteristic of environmental epidemiology is the multi-dimensional aspect of exposure patterns, frequently reduced to a cumulative exposure for simplicity of analysis. By adopting a flexible Bayesian clustering approach, we explore the risk function linking exposure history to disease. This approach is applied here to study the relationship between different smoking characteristics and lung cancer in the framework of a population based case control study.

**Methods:**

Our study includes 4658 males (1995 cases, 2663 controls) with full smoking history (intensity, duration, time since cessation, pack-years) from the ICARE multi-centre study conducted from 2001-2007. We extend Bayesian clustering techniques to explore predictive risk surfaces for covariate profiles of interest.

**Results:**

We were able to partition the population into 12 clusters with different smoking profiles and lung cancer risk. Our results confirm that when compared to intensity, duration is the predominant driver of risk. On the other hand, using pack-years of cigarette smoking as a single summary leads to a considerable loss of information.

**Conclusions:**

Our method estimates a disease risk associated to a specific exposure profile by robustly accounting for the different dimensions of exposure and will be helpful in general to give further insight into the effect of exposures that are accumulated through different time patterns.

## Background

Multi-dimensional exposure patterns are ubiquitous in environmental epidemiology. Typically, full exposure history is collected for each study participant, aimed primarily at recording a measure of intensity of exposure for each relevant period of time. Integrating such time dependent exposure patterns into a model of risk is a classical challenge frequently encountered by epidemiologists [1-5].

The simplest commonly used approach to summarise exposure history is to compute the cumulative life time exposure (e.g. pack-years for smokers or work-life exposure to known occupational carcinogens). This straightforward index of cumulative exposure essentially reduces a complex time pattern to a one dimensional summary, making strong assumptions on the equivalence of the roles of intensity and duration, an assumption that has been questioned as too simplistic by several authors, for example in the context of smoking and lung cancer [6,7].

Here, we present a novel statistical approach for this task, based on a flexible semi-parametric Bayesian approach, and demonstrate its utility for assessing the effects of different dimensions of exposure (intensity, duration and delay since the end of exposure). For this proof of principle, we have chosen the context of a strong and well established relationship, namely smoking and lung cancer.

As exemplified in the case of smoking, detailed exposure profiles generally consist of a vector of recorded characteristics, either continuous or categorical, that aim to capture the full extent of the exposure history as completely as possible. However, to statistically analyse such data, the use of classical multivariate regression techniques can lead to unstable results, as there typically exists strong multi-collinearity between the variables that make up the exposure profile. In addition, classical parametric models based on linear combinations of predictor variables make strong assumptions of additivity of effects on the log scale, assumptions that are thought to be biologically unrealistic. In this context, it is thus of great interest to propose flexible approaches going beyond the logistic model with linear combinations of covariates.

Partition and clustering methods are semi-parametric approaches that aim to discretise a multi-dimensional risk surface into cells having similar risks; well known examples of such approaches are the Classification and Regression Trees (CART) [8] or Multifactor Dimensional Reduction (MDR) [9] methods. However, for such hard clustering methods, the grouping is fixed and is sensitive to tuning parameters and initialisation and can neglect the inherent uncertainty associated with partitioning, with the consequence that the variability of the risk is underestimated.

In this paper, we propose drawing on Bayesian clustering approaches to approximate the risk function, linking the exposure history to the disease. Broadly speaking, our formulation partitions the exposure characteristics into clusters and links these to the disease response in a unified Bayesian model that we refer to as profile regression [10]. Profile regression has been used in environmental epidemiology [11] as well as for looking for gene-gene interactions [12]. Here, we build on this work to demonstrate how profile regression can be used to derive a multi-dimensional risk estimate, leading to a better understanding of the important drivers of the risk. We explore the sensitivity of our model and illustrate its performance with respect to standard logistic analysis and CART in a small simulation study and in our case study. Additionally we compare the risk estimates produced by the clustering model with those corresponding to the standard summary index pack-years.

## Methods

### Data

The ICARE study, conducted from 2001 to 2007, is a large multicentre population-based case-control study of respiratory cancers. The study was approved by the Institutional Review Board of the French National Institute of Health and Medical Research (IRB-Inserm, no. 01-036), and by the French Data Protection Authority (CNIL no. 90120). Each subject gave a written and informed consent. In order to protect the confidentiality of personal data and to fulfil legal requirements, the questionnaire included only an identification number, without any nominative information. The same identification number was used for biological specimen. The link between the name and the identification number (to the exclusion of any other data) was kept by the cancer registry of the area where the subject was interviewed. Further details have been described previously [13].

The present analysis focused on men with lung cancer and their population controls, restricting the dataset to 4,658 males with full smoking histories. Of these 1,995 are cases. We also separately consider the male dataset stratified by histological cell type. For the histological analyses, we use all the controls, but only the relevant cases, resulting in datasets of size 3,365 for adenocarcinoma (702 cases), 3,359 for squamous (696 cases) and 2,933 for small cell cancers (270 cases). The smoking covariates that we study are: intensity (cigarettes per day), duration (years as a smoker), time since smoking cessation (years) and pack-years. For each covariate we categorise the data into 5 categories (summarised in Table 1), chosen to contain approximately balanced numbers of individuals as well as being easily interpretable.

**Table 1:** Summary of covariate categories

**Covariate**	**Category id**	**Category description**	**N. Subjects**
Average intensity of smoking	0	Non-smoker	823
	1	0 < cigarettes per day ≤ 10	716
	2	10 < cigarettes per day ≤ 20	1540
	3	20 < cigarettes per day ≤ 30	1014
	4	30 < cigarettes per day	550
	NA	Not available	15
Duration of smoking	0	Non-smoker	823
	1	0 < years ≤ 20	972
	2	20 < years ≤ 30	887
	3	30 < years ≤ 40	1073
	4	40 < years	903
Time since quit smoking	0	Non-smoker	823
	1	20 < years	870
	2	10 < years ≤ 20	583
	3	0 < years ≤ 10	996
	4	Current smoker	1386
Pack-years	0	Non-smoker	823
	1	0 < pack-years ≤ 15	1089
	2	15 < pack-years ≤ 30	1043
	3	30 < pack-years ≤ 45	888
	4	45 < pack-years	800
	NA	Not available	15

The categories used to apply profile regression to data from the ICARE case-control study.

Within our model we adjust for age, education level, whether the subject has ever worked in a job known to entail exposures associated with lung cancer (i.e. List A [14]), and for the centre where the data was collected. These adjustments are done by treating the variables as fixed effects as described in the statistical model section below.

### Statistical background

In order to explore the associations between smoking characteristics (the covariates) and the risk of lung cancer (the outcome), most common methods attempt to perform a direct regression of the outcome against the covariates. In contrast, our proposed method uses an alternative approach, based upon a statistical mixture model designed to flexibly group individuals into clusters, allowing the clusters to be jointly determined by both covariates and outcomes. By then looking at typical cluster characteristics, in particular the probabilities of covariate values (which we call the profile) for any particular cluster, alongside the average risk of disease for that cluster, we can draw conclusions about patterns within the profile that appear to be related to increased or decreased risk.

As a specific simplified example of how such a model might be used, suppose we fit the model to a subset of the smoking covariates (intensity, duration and time since cessation). In the resulting analysis, imagine that the subjects were split into three clusters. Suppose cluster 1 is identified as having a high risk for the disease, cluster 2 contains subjects at average risk and cluster 3 consists of subjects at low risk. By looking at the average profile in the high risk cluster 1, we might see for example a higher than average probability of being in the highest intensity category, the longest duration category and a raised probability of being a current smoker. Of course, if the method resulted only in such simplified results, this would provide no insight beyond the well known harmful effects of tobacco smoke, but in practice we might hope for a larger number of clusters, covering a range of disease risks, each with different profiles, allowing us to tease out more subtle relationships between covariate combinations and risk.

### Model formulation

The underlying clustering model that we use is based on a Dirichlet process (DP) formulation, a well recognized semi-parametric technique that has been extensively studied [15,16] and which can be implemented using a Markov chain Monte Carlo (MCMC) algorithm. To formalise the ideas behind the method we employ, consider that we have *N* individuals, indexed by *i*. For each individual we have an observed disease outcome *y*_*i*_ and a covariate profile ***x***_*i*_=(*x*_*i*,1_,…,*x*_*i*,*J*_), consisting of the *J* covariates that we are interested in studying, where covariate *j* is one of *L*_*j*_ possible categories.

The model that we adopt is a joint probability model for the outcome *y*_*i*_ and profile ***X***_*i*_, where for each individual, independent of every other,

(1) $$p(Y_{i},X_{i}|\Theta )=\overset{\infty }{\underset{c=1}{\sum }}\psi_{c}p(Y_{i}|\Theta_{c},\Theta_{0})p(X_{i}|\Theta_{c},\Theta_{0}).$$

This describes an infinite mixture model, where the weight of mixture component *c* is given by *ψ*_*c*_, and, for each component, the probability models for the outcome *y*_*i*_ and the profile ***X***_*i*_ are independent, *conditional* on some component specific parameters ***Θ***_*c*_ and some global parameters ***Θ***_0_. In the left hand side we summarise the complete set of parameters as ***Θ***=(***Θ***_0_,*ψ*_1_,***Θ***_1_,*ψ*_2_,***Θ***_2_,…). In order to make inference, it is convenient to introduce the additional allocation parameter *Z*_*i*_, with the interpretation that *Z*_*i*_=*c* indicates that individual *i* is assigned to mixture component *c*. If the prior allocation probabilities are given by *p*(*Z*_*i*_=*c*)=*ψ*_*c*_, posterior inference on ***Z***=(*Z*_1_,*Z*_2_,…,*Z*_*N*_) then provides us with information on the groupings, or clustering, of the individuals.

The mixture weights ***ψ***={*ψ*_*c*_,*c*≥1} are modeled according to a “stick breaking” representation [17] of a Dirichlet process prior using the following construction. We define a series of independent random variables *V*_*j*_, each having distribution *V*_*j*_∼Beta(1,*α*). This generative process is referred to as a stick-breaking formulation since one can think of *V*_1_ as representing the breakage of a stick of length 1, leaving a remainder of (1−*V*_1_) and then a proportion *V*_2_ begin broken off leaving (1−*V*_1_)(1−*V*_2_) etc. More details about this construction are given in Additional file 1: Appendix 1 in the supplemental material.

The flexibility of this model is provided by the choices for the response sub-model $p(Y_{i}|Z_{i},\Theta_{Z_{i}},\Theta_{0})$ and the profile sub-model $p(X_{i}|Z_{i},\Theta_{Z_{i}},\Theta_{0})$. For the response sub-model, we assume $y_{i}|Z_{i},\Theta_{Z_{i}},\Theta_{0}∼\text{Bernoulli}(\pi_{i})$ where $\text{logit}(\pi_{i})=\theta_{Z_{i}}+\beta^{\prime }w_{i}$. Here, *θ*_*c*_ is the log odds of disease for component *c* and ***w***_*i*_ are additionally observed fixed effects covariates or confounders for individual *i*, with regression coefficients ***β*** that do not depend upon the mixture component to which individual *i* is allocated.

For the profile sub-model, conditional upon the allocation *Z*_*i*_, we assume independence between covariates, such that $X_{i,j}|Z_{i}=c∼\text{Multinomial}(1,\phi_{Z_{i},j}),$ where $\phi_{c,j}=(\phi_{c,j,1},\phi_{c,j,2},\ldots ,\phi_{c,j,L_{j}})$ is the vector of probabilities associated with cluster *c* for each of the *L*_*j*_ possible categories that could be observed for covariate *j*.

Together these two sub-models define our component specific parameters ***Θ***_*c*_=(*θ*_*c*_,*ϕ*_*c*,1_,…,*ϕ*_*c*,*J*_) and the global parameters ***Θ***_0_=***β***.

Adopting a Bayesian perspective allows a natural way for making joint inference on the full set of parameters. Such an approach requires further specification of prior distributions for these parameters. We adopt similar priors to those used by Molitor et al. [10], using a conjugate approach where possible. A full specification can be found in Additional file 1: Appendix 1.

#### Inference

Because the posterior distribution resulting from these priors and the likelihood in model (1) is non-standard, we use a simulation based method and an MCMC sampler to make inference. Contrary to standard practice whereby a truncated version of model (1) [17-20] is typically considered, the new sampler (Hastie DI, Liverani S, Papathomas M, Richardson S: *PReMiuM, An R package for Profile Regression Mixture Models using Dirichlet Processes*, submitted) that we use here does not require any truncation a priori but relies on the introduction of a latent variable which allows a finite number of clusters to be sampled within each iteration of the sampler as specified for previous samplers of a similar nature [21-23]. This sampler uses a combination of Gibbs and Metropolis-within-Gibbs steps to sample from the infinite mixture (only retaining the parameters of a finite number of mixture components including all those to which individuals are allocated at each sweep). If there are missing values in the profile data, these can also be sampled within the MCMC sampler.

#### Post-processing

One way to summarise the characteristics of the posterior clustering from an MCMC run is to perform several post-processing steps [10]. In brief, a dissimilarity matrix is constructed that records *for each pair of individuals* the proportion of the MCMC iterations that they were allocated to different mixture components. Partitioning around medoids (PAM) [24] or using square error distance [25] is then performed on this dissimilarity matrix to determine a representative clustering. Using this representative clustering, the characteristics of its clusters arise from examining the MCMC output for the relevant parameters [10].

Any such representation of the rich output of the DP process is necessarily reductive and should not be over-interpreted as it is linked to the chosen way of postprocessing the dissimilarity matrix. Nevertheless, in our case study, we found that it provides a useful representation to understand better what dimension of exposure drives the risk.

#### Implementation

The implementation of profile regression was performed using the R package PreMiuM which is freely available from the R website (http://cran.r-project.org/web/packages/PReMiuM/). See Additional file 1: Appendix 2 for associated references and command lines.

### Quantifying patterns

Examining the typical profiles of clusters associated with different levels of risk can provide a hypothesis-generating descriptive exploration of potential associations between covariates and link these to the outcome. However, it is also of interest to quantify the roles of specific covariates. Fortunately, with little extra effort, our simulation based method allows such results to be derived, through the use of posterior predictions.

Suppose that we wish to understand the role of a particular covariate or group of covariates. We can specify a number of predictive scenarios (pseudo-profiles), that capture the range of possibilities for the covariates that we are interested in. For each of these pseudo-profiles we can see how these would have been allocated in our mixture model to understand the risk associated with these profiles. More details on the pseudo profiles are available in Additional file 1: Appendix 3.

To illustrate, consider our simple example above, where the smoking covariates under study are intensity, duration and time since cessation. Suppose further that we have a simplified categorical structure for each variable, with each individual being categorised into 0=non-smoker, 1=Low, 2=Medium or 3=High for each of these covariates. If we are particularly interested in how intensity affects the risk, we can set up the following pseudo-profiles for (*x*_INT_,*x*_DUR_,*x*_TSC_): the non-smoker (0,0,0), the low intensity smoker (1,NA,NA), the average intensity smoker (2,NA,NA) and the high intensity smoker (3,NA,NA). The non-smoking pseudo-profile is included for reference, so that we can compute the odds ratio (OR) with respect to this profile for each of the other pseudo-profiles. Notice that for the intensity profiles, the other variables (duration and time since cessation) are treated as missing (denoted by NA). We discuss the technicalities of this in Additional file 1: Appendix 4.

As an output of our method, for each of our non-smoker and low, medium and high intensity pseudo-profiles, we can compute the probabilities that the pseudo-profile belongs in each cluster. These probabilities do not affect the fit of the model, which is determined wholly by the observed data. However, with these probabilities we can construct a cluster-averaged estimate of the log odds for each particular pseudo-profile. This is repeated at each stage of our model fitting process resulting in a density of these log odds (or the log odds ratio with respect to the non-smoking reference pseudo-profile) that gives us an estimate of the effect of the particular pseudo-profile. This can be compared to other pseudo-profiles, allowing us to derive a better understanding of the role of specific covariates.

## Results and discussion

### Overall patterns

**Figure 1:**
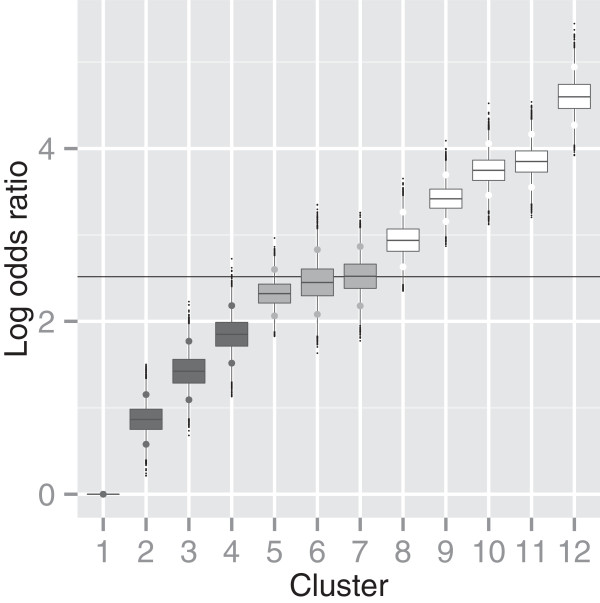
**Log odds ratios of clusters.** Log odds ratio relative to the non-smoking cluster 1, for the clusters in the representative clustering of the analysis with intensity, duration, time since cessation and pack years.

Our first analysis concentrates on the four primary smoking variables, intensity, duration, time since cessation and pack-years. Results are presented in Figure 1 and Table 2.

**Table 2:** Summary of cluster profiles

**Cluster**													
		**1**	**2**	**3**	**4**	**5**	**6**	**7**	**8**	**9**	**10**	**11**	**12**
No. Subjects		823	748	212	204	516	103	158	159	570	386	354	425
Log OR		0	0.87	1.43	1.85	2.32	2.45	2.52	2.94	3.42	3.75	3.85	4.6
INT	0	1.00	0.00	0.00	0.00	0.00	0.01	0.01	0.00	0.00	0.00	0.00	0.00
	1	0.00	0.47	0.83	0.01	0.03	0.01	0.90	0.08	0.01	0.00	0.00	0.00
	2	0.00	0.41	0.13	0.25	0.70	0.01	0.07	0.90	0.96	0.03	0.03	0.15
	3	0.00	0.11	0.02	0.57	0.25	0.30	0.01	0.01	0.01	0.92	0.25	0.46
	4	0.00	0.01	0.01	0.16	0.01	0.67	0.01	0.00	0.00	0.04	0.72	0.38
DUR	0	1.00	0.00	0.00	0.00	0.00	0.01	0.01	0.00	0.00	0.00	0.00	0.00
	1	0.00	0.90	0.13	0.83	0.13	0.19	0.02	0.01	0.00	0.01	0.01	0.00
	2	0.00	0.09	0.52	0.15	0.84	0.65	0.08	0.04	0.01	0.22	0.18	0.01
	3	0.00	0.01	0.29	0.01	0.02	0.14	0.30	0.70	0.51	0.62	0.63	0.15
	4	0.00	0.00	0.06	0.01	0.01	0.02	0.59	0.24	0.48	0.14	0.19	0.84
TSC	0	1.00	0.00	0.00	0.00	0.00	0.01	0.01	0.00	0.00	0.00	0.00	0.00
	1	0.00	0.66	0.21	0.51	0.24	0.39	0.04	0.04	0.02	0.04	0.05	0.00
	2	0.00	0.16	0.26	0.24	0.22	0.22	0.07	0.19	0.10	0.14	0.16	0.04
	3	0.00	0.08	0.19	0.16	0.22	0.20	0.36	0.30	0.33	0.40	0.32	0.38
	4	0.00	0.09	0.34	0.09	0.31	0.19	0.53	0.46	0.55	0.42	0.46	0.58
PY	0	1.00	0.00	0.00	0.00	0.00	0.01	0.01	0.00	0.00	0.00	0.00	0.00
	1	0.00	0.97	0.91	0.07	0.12	0.01	0.58	0.02	0.00	0.00	0.00	0.00
	2	0.00	0.03	0.07	0.91	0.87	0.02	0.39	0.67	0.34	0.01	0.00	0.00
	3	0.00	0.00	0.01	0.01	0.01	0.76	0.01	0.30	0.65	0.90	0.07	0.02
	4	0.00	0.00	0.00	0.00	0.00	0.20	0.01	0.01	0.01	0.08	0.92	0.97

Table of distribution mean for characteristics of clusters from the representative clustering of the analysis of Intensity, Duration, Time Since Cessation and PackYears. For the covariates, the distribution is of the probability that the covariate is in each category.

Using post-processing as described above to form a representative clustering, the population is split between 12 clusters. Figure 1 shows a box-plot of the posterior distribution for the log odds ratio (relative to the lowest risk cluster 1) for the 12 clusters, showing in particular four clusters with increasing risk (i.e. having a 95% Credibility Interval CI for the log odds ratio relative to the lowest risk cluster - marked by the larger points - containing only values above 1).

Table 2 summarises the posterior mean of the associated profile probabilities. We can observe immediately that the lowest risk cluster 1 is made up exclusively of non-smokers. Examining the patterns in this table suggests that high categories of smoking duration found in our study are more influential than high categories of intensity. The highest risk cluster 12 is associated with the highest duration category, but primarily with category 3 for intensity, whereas the cluster with the highest intensity, cluster 11, is associated with a lower odds ratio (3.85) than cluster 12 (4.6). Providing further support of this pattern, individuals in cluster 9, which are largely in intensity category 2 but have still a high log odds ratio (3.42, relative to the lowest risk cluster) as they are also associated with high probabilities of long smoking duration.

### Predictive log OR for different combinations of intensity and duration of smoking habits

Whilst selecting a representative clustering may highlight some interesting patterns, focussing on a single clustering is limited in scope. It is perhaps of more interest to consider and contrast various pseudo-profiles of covariate patterns, allowing us to better understand the role of each of the covariates.

**Figure 2:**
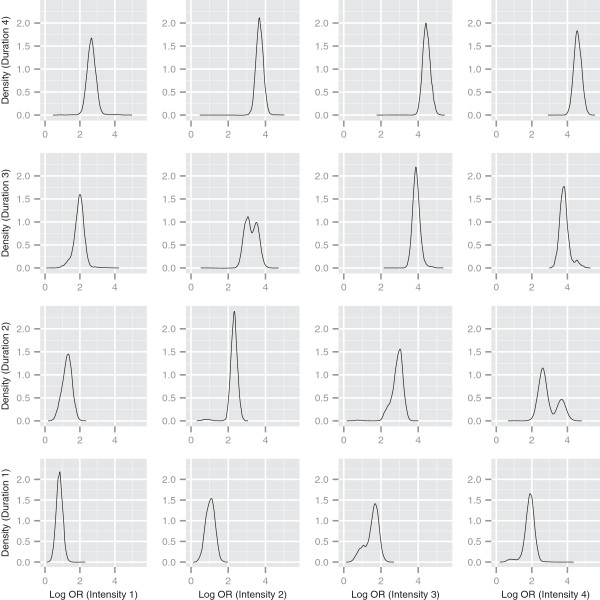
**Log odds ratios for intensity and duration combinations.** Density estimates of predicted log odds ratios relative to a non-smoking profile, for different intensity and duration combinations. Time since cessation and pack years treated as missing in pseudo-profiles.

In Figure 2, we plot 16 curves, corresponding to the posterior predictive densities of the log odds ratio (relative to a non-smoking pseudo-profile) for combinations of the 4 (smoking) categories of intensity and duration. For the pseudo-profiles plotted, both time since cessation and pack-years were treated as missing, meaning that these variables do not contribute to which cluster these pseudo-profiles are allocated. These density plots allow us to separate out effects of intensity and duration on risk, and to visually understand how the log odds ratio changes as we alter these covariates. Our initial observation is that the posterior distribution of risk is wider for some combinations of exposures than others, reflecting that there is less information in the data on these specific patterns. In a few cases, these densities are bimodal or skewed reflecting uncertainty in the allocation of the particular combination between clusters with markedly different risks. As we might expect, increasing intensity (moving to the right along a row of plots) and increasing duration (moving up along a column of plots) results in a shift of the density to higher log odds ratios. This is particularly apparent when looking at the posterior mean log odds ratio for each combination (see Table 3), which ranges from 0.83 for the pseudo-profile with intensity and duration in the lowest smoking categories, to 4.58 for the pseudo-profile with intensity and duration in the highest smoking categories.

**Table 3:** Pseudo-profile log odds ratio distribution summaries

**Intensity**	**Duration**	**Log odds ratio**	**Pack-years**
1	1	0.83 (0.19)	1.02 (0.02)
	2	1.26 (0.28)	1.06 (0.04)
	3	1.95 (0.32)	1.33 (0.16)
	4	2.66 (0.29)	1.47 (0.20)
2	1	1.03 (0.24)	1.18 (0.14)
	2	2.29 (0.26)	1.87 (0.12)
	3	3.24 (0.33)	2.52 (0.22)
	4	3.69 (0.21)	2.91 (0.12)
3	1	1.52 (0.37)	1.70 (0.34)
	2	2.87 (0.33)	2.45 (0.25)
	3	3.90 (0.21)	3.21 (0.12)
	4	4.45 (0.21)	3.75 (0.10)
4	1	1.86 (0.32)	2.22 (0.35)
	2	2.92 (0.54)	3.44 (0.31)
	3	3.83 (0.29)	3.94 (0.04)
	4	4.58 (0.22)	3.95 (0.05)

**Figure 3:**
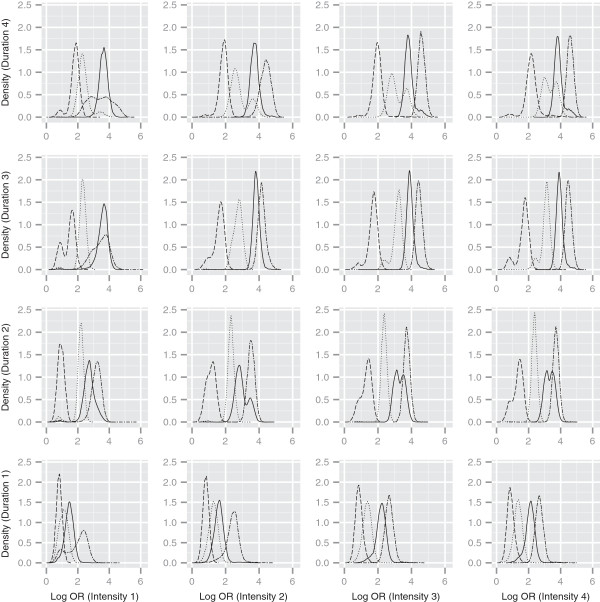
**Log odds ratios for intensity, duration and time since cessation combinations.** Density estimates of predicted log odds ratios relative to a non-smoking profile, for different intensity and duration combinations. Line types denote different time since cessation categories: dashed=1, dotted=2, solid=3, and double dashed=4. Pack-years treated as missing in pseudo-profiles.

Posterior distribution means (standard deviations) for 16 combinations of intensity and duration, where time since cessation and pack years are missing, corresponding to the distributions in Figure 3. Also shown for each combination is the mean (standard deviation) of expected the pack years category.

We might reasonably ask what the impact is of the other smoking profile variables that we treat as missing in the above plots. To gain some idea of the *typical* pack-year value associated with each pseudo profile, we can treat the categories in an ordinal way, and use the allocation probabilities and the pack-year profile probabilities to compute an *expected pack-year category value* associated with each pseudo-profile. The associated average pack-year expected category value for each pseudo-profile is also tabulated in Table 3 for each pseudo-profile, along with the standard deviation. Most interesting in these values is the observation that although the expected pack-years category is higher for the pseudo-profile with intensity category 4 and duration category 2 than for the pseudo-profile with intensity category 3 and duration category 3 (3.44 compared to 3.21) the posterior mean log odds ratio is considerably lower (2.92 compared to 3.90).

### Sensitivity, comparative evaluation and model fit

#### 

##### Sensitivity

In this section, we explore how “sensitive” profile regression is to changes in its implementation.

The ability of the Dirichlet process model to fit a dataset is clearly dependent on the number of components allowed by the prior structure. In fact, the implied prior on the number of clusters in the partition of the Dirichlet Process is completely determined by the size of the sample *N* and the parameter *α*[15,26]. Liu [27] showed that given a fixed *α*, $E[\;K|\alpha ,N]\approx \alpha \text{log}(1+\frac{N}{\alpha })$.

All the results presented in this paper are obtained with *α*=1. In order to investigate the impact of the priors on *α* in our analysis, we applied profile regression to our data with different values of *α* (3.6 and 10 : these values have been studied previously in other epidemiological contexts [10,26]).

As expected the two new representative clusterings (each with 13 clusters) are slightly different from that obtained with *α*=1, with one additional cluster. However, as can be seen in of Additional file 1: Figure S3, which displays the box-plots of the posterior distribution of the log OR for the two new representative clusterings, the ranges obtained with *α*=3.6 (0.87 to 4.38) and *α*=10 (0.89 to 4.60) remain similar to those presented previously in Table 2 (0.87 to 4.60). By examining in more detail the clusters forming these three representative clusterings, we see that the low and high end clusters are stable and that splitting among the average risk groups has led to an additional cluster.

Whilst comparing the different clusterings may provide some insights on how these changes on *α* could affect our results, we should emphasize again that comparison between representative partitions should not be over-interpreted. It is perhaps more relevant to check how these changes affect the conclusions drawn for the pseudo-profiles. We present in Additional file 1: Figure S4 the three posterior predictive densities of the log OR with *α*=1,3.6,10. The densities are almost coincident, which suggests that the risk predictions derived from our approach are not sensitive to choice of *α*. Combining these observations, we conclude that although the value of *α* has a “small” effect on the representative clustering, the epidemiological conclusions regarding the role of the covariates seem unaffected.

Our next concern was to investigate whether a different discretisation of the smoking covariates affects the conclusions drawn from our model. We thus applied profile regression with a different meaningful categorisation of the exposure information (the new discretisation is summarised in Additional file 1: Table S1). Admittedly, the population is split this time between fewer clusters (10 instead of 12), but examining the box-plot of the posterior distribution for the log odds ratio displayed in Additional file 1: Figure S5 we can see immediately that the range of the new values of the log OR (0.72 to 4.41) is similar to that obtained with the previous finer categorisation (0.87 to 4.60).

An indicative comparison of the full profiles associated with the new representative clustering can be derived from the posterior mean of the associated profile probabilities, tabulated in Additional file 1: Table S2. This table supports the same conclusions as those drawn with the previous discretisation: high categories of duration appear to carry a greater risk than high categories of intensity (e.g. comparing cluster 9 and 10). Further comparison is provided in Additional file 1: Figure S6 which plots the posterior predictive densities of the log OR for the corresponding pseudo-profiles. As expected, with fewer categories, the cells are less sparse reducing uncertainty, and the density plots are all unimodal.

This detailed examination of the results indicates that a different discretisation leads, as expected, to a different representative clustering but that the epidemiological conclusions about the pattern of risk remain similar.

##### Comparative evaluation

Classification and regression tree methods (CART) are the most commonly used non-parametric methods that require no distributional assumptions. CART uses tree building methods, a form of binary recursive partitioning, and classifies subjects or predicts the outcome by selecting the most important risk factors available from the study population. This method is becoming more widely used in cancer research [28-31]. Logistic regression is the standard approach for estimating the association between a disease and epidemiological risk factors. A comparison of profile regression, logistic regression and CART has been carried out previously in another epidemiological context [11], in which CART was found to perform slightly better.

To investigate further the relative advantages of these methods, we carried out a small simulation study corresponding to a case-control set-up with ten binary covariates representing, for example, environmental factors. The goal of this simulation exercise is to illustrate the behaviour of these methods in two contrasting scenarios, the first one corresponding to a logistic model with interactions, the second one to a situation with true contrasting profiles. We give full details in Additional file 1: Appendix 5 of how our simulated data were designed and what model validation criteria were used to assess the performance of the three compared approaches.

Additional file 1: Table S3 shows that profile regression has an overall good performance, even in the first scenario that directly appeals to a logistic set up, and is competitive with CART in the second scenario. It appears that the ability of profile regression to reveal apparent complex effects does not come at a cost for the goodness of fit in simpler set ups. Further details about this comparison are provided in Additional file 1: Appendix 5.

##### Model fit

To check how well these models fit the lung cancer data, we report in Table 4 a comparison of profile regression with CART [8] using logistic-type residuals [11]. We cannot directly compare our profile approach to logistic regression due to the perfect collinearity between non smoking status and the zero category for the discretised smoking covariates such as intensity and duration. In our lung cancer study, profile regression shows an improved performance with respect to CART. This is the case even if the latter has as many as 55 branches compared to the more interpretable representable partition with 12 clusters found by profile regression, which can be further visualised in Additional file 1: Figure S7. More details about this comparison are provided in Additional file 1: Appendix 6.

**Table 4:** Comparison of profile regression and CART

	**RMSEy**	**MAEy**	**Misclassification error**
profile regression	0.39	0.32	0.22
CART	0.42	0.34	0.24

Comparison of measures of fit for profile regression and CART on the ICARE data set.

### Accounting for other dose characteristics

It is also possible to use multiple curves on each sub-plot to look at the combined effect of 3 covariates. Figure 3 demonstrates this idea, with different line types correspond to different categories of time since cessation of smoking (only pack years in now treated as missing). As the time since cessation reduces (the dashed lines representing ex-smokers with long cessation time compared to the double dashed lines representing current smokers) the posterior mass moves to higher log odds ratio values. However, this shift is not uniform across all intensity/duration combinations. For example, when intensity and duration are both in the third category, there is a clear separation between all 4 categories of time since cessation. In contrast, when the duration is in category 3, and intensity is in category 1, the densities corresponding to the 2 highest time since cessation categories are almost coincident.

### Histological subtypes

**Figure 4:**
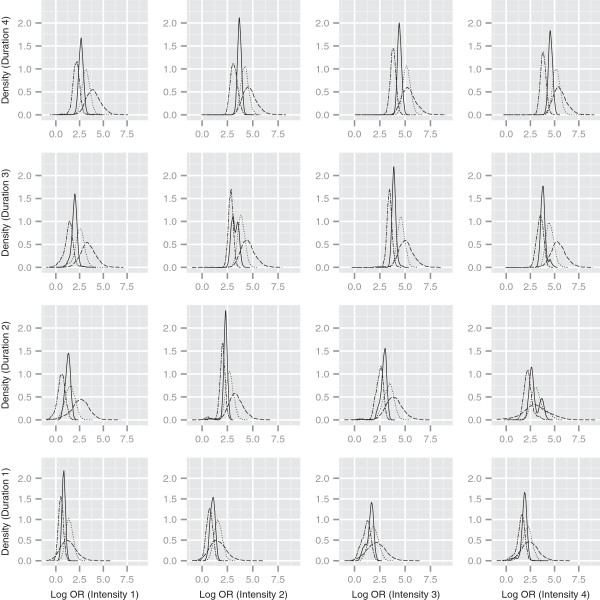
**Log odds ratios for different histologies.** Density estimates of predicted log odds ratios relative to a non-smoking profile, for different intensity and duration combinations. Line types denote different histologies: solid = all histologies, double dashed = adenocarcinoma, dotted = squamous and dashed = small cell. Time since cessation and pack-years treated as missing in pseudo-profiles.

We extend our analysis of intensity and duration to different histologies in Figure 4. In particular we predict the risk for 16 pseudo-profiles resulting from crossing the categories of these covariates for each of the different histological types (time since cessation and pack-years are both treated as missing). The densities are clearly different, demonstrating that small cell cancers appear to have a significantly higher log odds ratio (relative to the non-smoker) than adenocarcinomas. There is also some evidence to suggest that the risk of the small cell cancers increases at a faster level as duration and intensity increase.

## Conclusions

In this paper, we have used a novel statistical approach to provide further epidemiological insights into the relative effect of different type of smoking patterns on lung cancer. We demonstrated the power of our method for dissecting the effect of correlated exposure components on risk prediction, although the resulting inference cannot be interpreted in a causal manner. We will first discuss the statistical approach used in this analysis and then review its benefits in the framework of epidemiological understanding of correlated exposure patterns.

Using a flexible semi-parametric approach, we were able to partition a European smoking population into 12 typical groups corresponding to different combinations of smoking profiles associated with log odds ratios for lung cancer up to around 4 (relative to non-smokers). This range of risk is as expected [32] and previously observed in other studies [33]. Based on the three most important characteristics of smoking exposure (i.e. intensity, duration, time since cessation) as well as the cumulative pack-years, a representative partition of the risk surface was derived, composed of a number of typical smoking profiles that can be easily interpreted. By using a fully Bayesian semi-parametric technique, the predictive risk distributions appropriately incorporate the uncertainty in the partitioning. The resulting patterns are not necessarily varying linearly with the risk, which makes our approach useful when the relationship between a set of covariates and the disease risk is not of a standard functional form.

The profile regression method should not be seen as an approach aimed at replacing classical logistic regression but rather as a complementary approach. One of the main advantages of this model is the added flexibility it offers to disentangle the role of the different dimensions of exposure to any agent. Through the use of pseudo-profiles combining different exposure (e.g. smoking) characteristics, the method facilitates a quantitative understanding of what dimensions of exposure drive the disease risk, providing additional insight to that provided by a classical analysis using logistic regression, which is the reference method to demonstrate the importance of each predictor. Although other methods such as CART are also able in principle to capture similar patterns, we showed on our population that the high number of branches given by CART was not interpretable, in contrast to the reasonable number of clusters derived through post-processing of the dissimilarity matrix induced by our flexible method.

Whatever the approach used, it is always important to check sensitivity to various choices. In our case, the number of clusters has a direct relationship with the parameter *α*, and it is good practice to investigate the influence of different choices for *α*. We found that varying *α* in a reasonable range has only a small impact on the representative clustering obtained for our data. More importantly, we found stable results for the posterior predictive densities of the log odds ratio, which were hardly influenced by these changes in *α*. Another analysis choice is the level of discretisation of the covariates. Clearly, a compromise has to be struck between the degree of discretisation of continuous covariates and the size of the corresponding cells.

Starting with a finer discretisation gives more modelling flexibility but using an unnecessary large number of parameters may also lead to more instability. We applied our approach to two different discretisations of our exposure data and found similar conclusions in terms of shifts in the posterior distribution of the log odds as smoking intensity and duration increase. As expected, the finer discretisation leads to increased uncertainty for some combinations, highlighting that, in this case, the information in the data on some of the covariate patterns may be sparse.

We provide a freely available R package PreMiuM to implement our approach. This package can also cluster vectors of continuous exposures, but it cannot accommodate, at present, mixtures of categorical and continuous covariates (as would be required if our analysis included intensity and duration as continuous covariates).

Epidemiological analysis is often faced with the need to estimate risks associated with multicomponent exposure data. This has often led to reductive exposure summaries based on a compromise between pragmatic choice and subject specific etiological understanding. Our proposed approach offers a statistically principled way forward where information between similar individuals is shared and uncertainty is acknowledged.

In a first step we have explored the respective roles of intensity and duration using 16 pre-determined contrasting profiles. The shift of the predictive density to the right for each duration category, more marked than the shift observed in each row, highlights the importance of duration when compared to intensity as previously shown in epidemiological studies [3]. This should be interpreted with regards to the range for the high categories of duration (> 40 years) and intensity (> 30 cig/day) found in our study. Moreover, we notice that the predictive log OR hardly changes between the two highest categories of intensity risk when duration is at its highest, compatible with the levelling off for the risk at high intensity which has been reported in some studies [32]. Interestingly, we also notice that the pack-years do not capture the subtle differences between certain combinations corresponding to different risks. We were also able to investigate the effect of time since cessation of smoking. Our results suggest a potential differential effect of this delay whether smokers were light or heavy consumers.

Our ability to quantify the association for predetermined smoking profiles was extended to the different cancer subtypes. Figure 4 clearly highlights that the effect of smoking patterns is systematically higher for small cell lung cancer than adenocarcinomas, whatever the intensity and duration combination. There is also an indication that smoking duration is important for differentiating these histological types.

In principle our method allows consideration of a large number of components of exposure. In our particular case, we have also explored the respective role of dark versus light tobacco and filtered versus non filtered and were not able to find notable differences. This could be in part due to the difficulty of accurately recording these further characteristics in a retrospective study as well as the high correlation between dark tobacco, non-filter tobacco and heavy-intensity smoking creating insufficient contrast. Beyond investigating multiple components of exposure to the same agent, this approach could be further extended to study the effect of life long exposure to multiple agents. Along similar lines, semi-parametric Bayesian approaches have also been proposed to analyse highly correlated multiple agents [34]. From a public health point of view, when faced with environmental exposures that can be accumulated through different time patterns, it is very important to identify the driving component of the risk; whether it is purely linked to total dose or whether small protracted doses carry similar or lower risk than peak or high intensity. This question is highly relevant for giving health protection guidelines in occupational settings or for environmental risk monitoring. We believe that our semi-parametric approach offers a novel and effective way of characterising such complex relations.

## Competing interests

The authors declare that they have no competing interests.

## Authors’ contributions

IS co-ordinated the ICARE study and provided the data upon which this study and our results are based. DIH and SL designed the PReMiuM software used to implement our method. LA implemented the studies into the method’s sensitivity and compared the method to other techniques. All authors have been significantly involved in drafting the manuscript and have read and approved the final manuscript.

## Authors’ information

Sylvia Richardson and Isabelle Stücker are joint last authors.

## Pre-publication history

The pre-publication history for this paper can be accessed here:

http://www.biomedcentral.com/1471-2288/13/129/prepub

## Supplementary Material

Additional file 1 **Supplemental material.** PDF file containing the eAppendices, eFigures and eTables. Click here for file
