# Supplementary material for: A semi-parametric approach to estimate risk functions associated with multi-dimensional exposure profiles: application to smoking and lung cancer
Source: BMC Med Res Methodol. 2013 Oct 23;13:129. doi: 10.1186/1471-2288-13-129 (PMC3827926; doi:10.1186/1471-2288-13-129)
Supplement: Additional file 1 — Supplemental material. PDF file containing the eAppendices, eFigures and eTables. [file 1471-2288-13-129-S1.pdf]

Supplemental Material – A semi-parametric approach to estimate risk functions associated with multi-dimensional exposure profiles: application to smoking and lung cancer

David I. Hastie, Silvia Liverani, Lamiae Azizi,
Sylvia Richardson, Isabelle Stücker

eAppendix 1

Throughout our analyses we use a stick breaking representation of a Dirichlet process prior which for $i = 1, \ldots, N$ can be written as:

$$\begin{aligned} Z_i|\boldsymbol{\psi} &\sim \sum_{c=1}^{\infty} \psi_c \delta_c(\cdot) \\ \psi_1 &= V_1 \\ \psi_c &= V_c \prod_{r<c} (1 - V_r) \quad c \geq 2 \\ V_j &\sim \text{Beta}(1, \alpha) \end{aligned}$$

where $\delta_x(\cdot)$ is the Dirac delta measure centred at x.

Additionally, throughout this paper we adopt the following priors for the other parameters in our model:

$$\begin{aligned} \theta_c &\sim \text{t}_7(0, 2.5), \quad c = 1, 2, \ldots \\ \boldsymbol{\phi}_{c,j} &\sim \text{Dirichlet}(1, \ldots, 1), \quad j = 1, \ldots, J, \;\; c = 1, 2, \ldots \\ \beta_r &\sim \text{t}_7(0, 2.5), \quad r = 1, \ldots, R, \end{aligned}$$

where $\mathrm{t}_m(l, s)$ denotes the Student's–t distribution, with m degrees of freedom, and location l and scale s and R is the number of fixed effect coefficients.

eFigure1 provides a directed acyclic graph (DAG) of our model.

eAppendix 2

The code for profile regression is freely available in package `PReMiuM`[e1] of the `R` statistical software[e2]. Once the data and the package have been loaded in `R`, the lines of code to implement profile regression are given below.

```
runInfoObj <- profRegr(yModel="Bernoulli", xModel="Discrete",
  covNames=covNames, alpha=1, nSweeps=10000, nBurn=10000,
  data=icare, output="output/output",
  fixedEffectsNames = fixedEffectsNames, predict=preds)
dissimObj <- calcDissimilarityMatrix(runInfoObj)
clusObj <- calcOptimalClustering(dissimObj, useLS=T)
riskProfileObj <- calcAvgRiskAndProfile(clusObj)
clusterOrderObj <- plotRiskProfile(riskProfileObj,"summary.png")
outputpredictions <- calcPredictions(riskProfileObj)
```

Additional flexibility of the code is provided by the choice of hyperparameters, samplers and initial number of clusters, which can be easily customised. Two criteria can be used to construct the representative clustering. Here, our results use the setting `useLS=TRUE`, which selects from among all the visited partitions, that which corresponds to the smallest square error distance to the dissimilarity matrix. Alternatively, `useLS=FALSE` (the default option) uses the Partitioning around medoids with its default implementation in `R` to return the corresponding representative clustering.

We run our sampler for 20000 iterations, discarding the first 10000. It is a well known problem for samplers of such models that due to the highly multi-modal nature of the clustering space, the sampler can sometimes struggle to move between equally well supported local modes, so that two separate runs may suggest different clusterings, and that this might be sensitive to the initial allocation of the chain. Guidance for the `PReMiuM` sampler suggests ensuring that the individuals are initially allocated into a larger number of clusters[e3] and we also use the marginal model posterior as a measure of convergence[e3].

eAppendix 3

At each sweep r of the MCMC sampler we can define an additional "allocation" variable, $\tilde{Z}_p^r$ corresponding to each pseudo-profile p. These variables do not affect the fit of the model, which is determined wholly by the observed data. However, for each pseudo-profile we can compute the posterior probabilities $p(\tilde{Z}_p^r = c|\boldsymbol{x}_p, \boldsymbol{\Theta}^r, D)$ where D is the observed data $(\boldsymbol{y}, \boldsymbol{x}_1, \ldots, \boldsymbol{x}_N)$. With these probabilities we can construct a cluster-averaged estimate of the log odds θ for each particular pseudo-profile at each sweep. Specifically,

$$\hat{\theta}_p^r = \sum_{c=1}^{\infty} p(\tilde{Z}_p^r = c|\boldsymbol{x}_p, \boldsymbol{\Theta}^r, D)\theta_c^r.$$

Looking at the density of these log odds (or the log odds ratio with respect to the non-smoking reference pseudo-profile) over MCMC sweeps gives us an estimate of the effect of a particular pseudo-profile, and can be compared to other pseudo profiles, allowing us to derive a better understanding of the role of specific covariates.

eAppendix 4

Unlike for the observed individuals where posterior allocation probabilities depend on both the profile and response sub-models, for the predictive pseudo-profiles the allocation probabilities are determined only by the profile sub-model. Furthermore, missing variables in the pseudo-profiles are ignored when the allocation probabilities are computed. The impact of this is subtle, but essentially means that the missing value will reflect the covariate patterns present in the main sample. For example, if high intensity is associated with high duration, the high intensity profile is likely to be assigned to a cluster which is also characterised by high duration, meaning that in effect, the missing duration would be treated as high duration for this profile. However, for a different pseudo-profile (e.g. low intensity), the missing value might be treated as something different (e.g. low duration). Because of this, the marginal effect of intensity that is derived has to be interpreted as a population average effect, over a population with similar characteristics to that under study.

eAppendix 5

We performed a small simulation exercise to illustrate the performance of profile regression, CART and logistic regression for predictive modelling, both when no apparent structure is present in the covariates and in the presence of a strong signal.

Scenario 1 We first generated 10 binary predictors $\mathbf{x} = (x_1, \ldots, x_{10})$ and a case-control disease status of 2500 unrelated subjects. Predictor variables were generated from a Bernoulli distribution with a probability of 0.5 and were randomly associated with the outcome y. Therefore, individual predictors were not statistically significant predictors of the outcome. We further considered two interaction terms, which were highly associated with the outcome. We considered all single predictors and two-way interactions to find the best logistic model. The multiple logistic approach

and stepwise selection procedure identified four single predictors and four interactions. Thus, the best logistic model is:

$$\log\left(\frac{\mathbb{P}(y=1)}{\mathbb{P}(y=0)}\right) = -0.81 + 0.20x_1 + 0.15x_2 + 0.23x_3 + 0.03x_4$$
$$+1.34x_1x_4 + 1.38x_2x_3 - 0.64x_1x_2 - 0.74x_3x_4$$

With the above model, we calculated the probabilities that a subject has a disease for given values of the predictors.

Scenario 2 Next, in order to examine the situation where the signal was stronger, we simulated a data set of sample size $N = 2500$ where individuals fell into one of three subgroups with the probabilities: $\psi_1 = 0.24$, $\psi_2 = 0.51$ and $\psi_3 = 0.25$ and we choose the probabilities that the covariates are in one of the 5 categories (these probabilities correspond to the $\phi_{c,j}$ in our approach) as shown in the eFigure 2 below. For each individual i we sampled a cluster $c_i \in \{1, 2, 3\}$ and, given these clusters, the vector of values for the 10 covariates using the $\boldsymbol{\phi}$ and $\boldsymbol{\psi}$ described above. We then generated $\beta_c \sim N(0, \sigma^2_\beta)$, calculated each individual i's true probability of disease as $p_i = p(y_i = 1|c_i, \mathbf{x}_i) = \text{expit}(\beta_0 + \beta_{c_i})$ and finally sampled the outcome $y_i \sim \text{Bernoulli}(p_i)$.

To check how well the different models fit the simulated data we used logistic "regression" type residuals and the misclassification error. Thus, we judged the quality of predictions by comparing predicted ("fitted") probabilities ($\hat{p}_i$) to the true probabilities (p_i) using the root mean square error given by $\text{RMSE}_p = \sqrt{N^{-1}\sum_{i=1}^{N}(\hat{p}_i - p_i)^2}$ and the mean absolute error given by $\text{MAE}_\text{p} = N^{-1}\sum_{i=1}^{N} \mid \hat{p}_i - p_i \mid$. We also compute similar quantities but with the generating probabilities p_i replaced the values of the observed outcome y_i. We denote these as RMSE_y and MAE_y respectively. The misclassification error criterion represents the proportion of the subjects misclassified as cases or controls.

In eTable 3 we give the measures of fit for profile regression, logistic regression and CART for both simulation studies. For Scenario 1, where the patterns are not clearly distinct, profile regression and logistic regression had similar performance with profile regression slightly better with respect to the MAE_y and the misclassification error. CART seems to struggle more to detect the interactions in this case with the worst performance among the three compared methods regarding all the measures of fit considered. For Scenario 2, where the patterns are more distinct than Scenario 1, the results suggest that profile regression is competitive with CART regarding prediction of the outcome or the true probabilities of disease. Logistic regression showed a reduction of power in results for this dataset.

eAppendix 6

We report a comparison of profile regression with CART on our real data. Due to the perfect collinearity present between smoking status and covariates for smokers when the latter covariates are discretised, we cannot directly compare to logistic regression. CART is available in the tree[e4] package in the R statistics software[e2]. The analysis using this method consists of three steps. First, a classification tree is built using standard recursive partitioning and a splitting rule. We use the gini criterion for this step, a criterion that minimises the heterogeneity of a group of subjects with respect to the outcome. At this point the maximal tree that has been produced probably overfits the data. The second step is pruning. We used 5-fold cross-validation and chose the tree that minimises the misclassification error. The resulting number of terminal nodes was 55, a result difficult to interpret. Moreover, CART requires datasets with no missing observations, so we based the comparison on a reduced dataset of 4643 subjects. We split our data into two groups. For one group, we sampled 2322 people from the population to train the models and the second group composed of the remaining 2321 people was used to measure the resulting models' prediction

errors.

eFigures

Figure 1: Directed Acyclic graph (DAG) for the profile regression model.

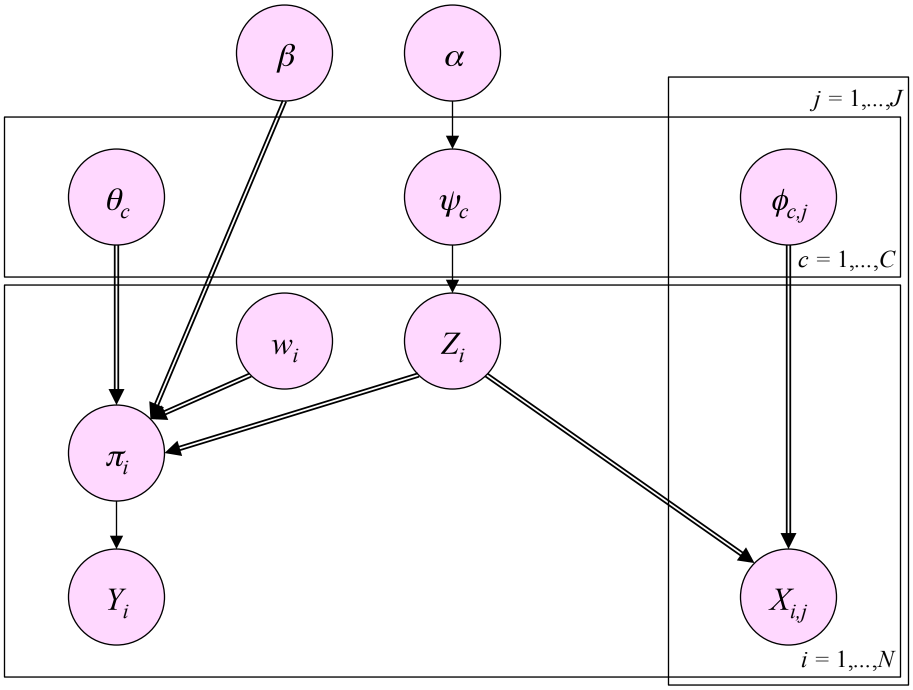

Figure 2: Probabilities that the 10 simulated covariates are in each category for scenario 2.

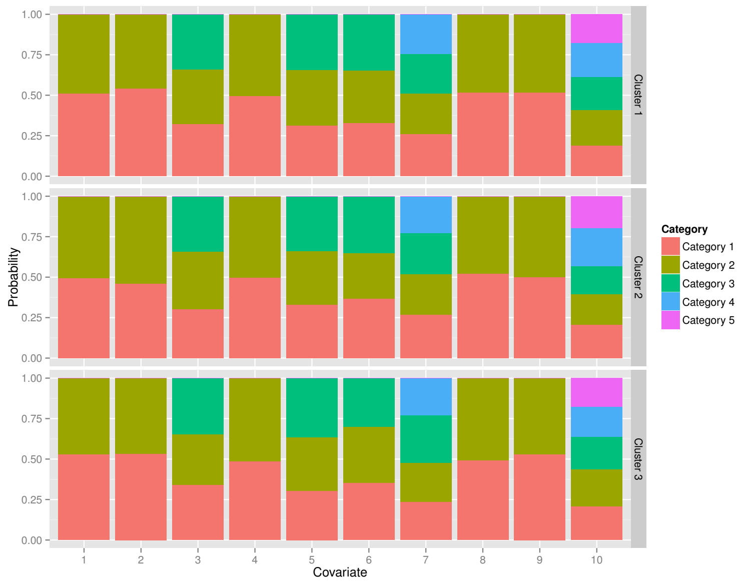

Figure 3: Log odds ratio relative to the non-smoking cluster 1, for the clusters in the representative clustering of the analysis with intensity, duration, time since cessation and pack years and with different values of α in profile regression: (a): $\alpha = 3.6$ and (b): $\alpha = 10$.

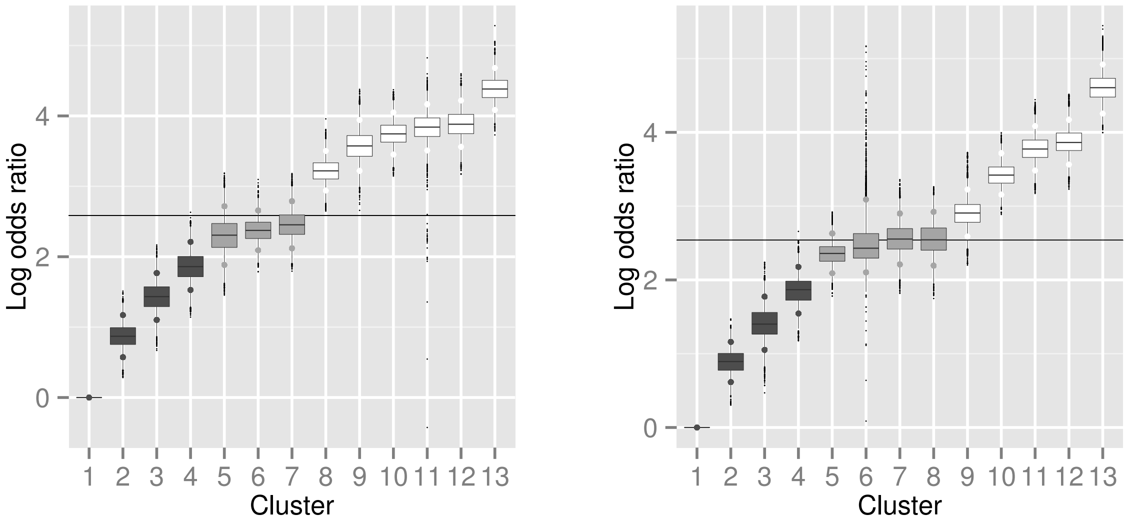

(a) (b)

Figure 4: Density estimates of predicted log odds ratios relative to a non-smoking profile, for different intensity and duration combinations with different values of α in profile regression: dotted= 1, dashed= 3.6 and double dashed = 10. Time since cessation and pack-years are treated as missing in pseudo-profiles.

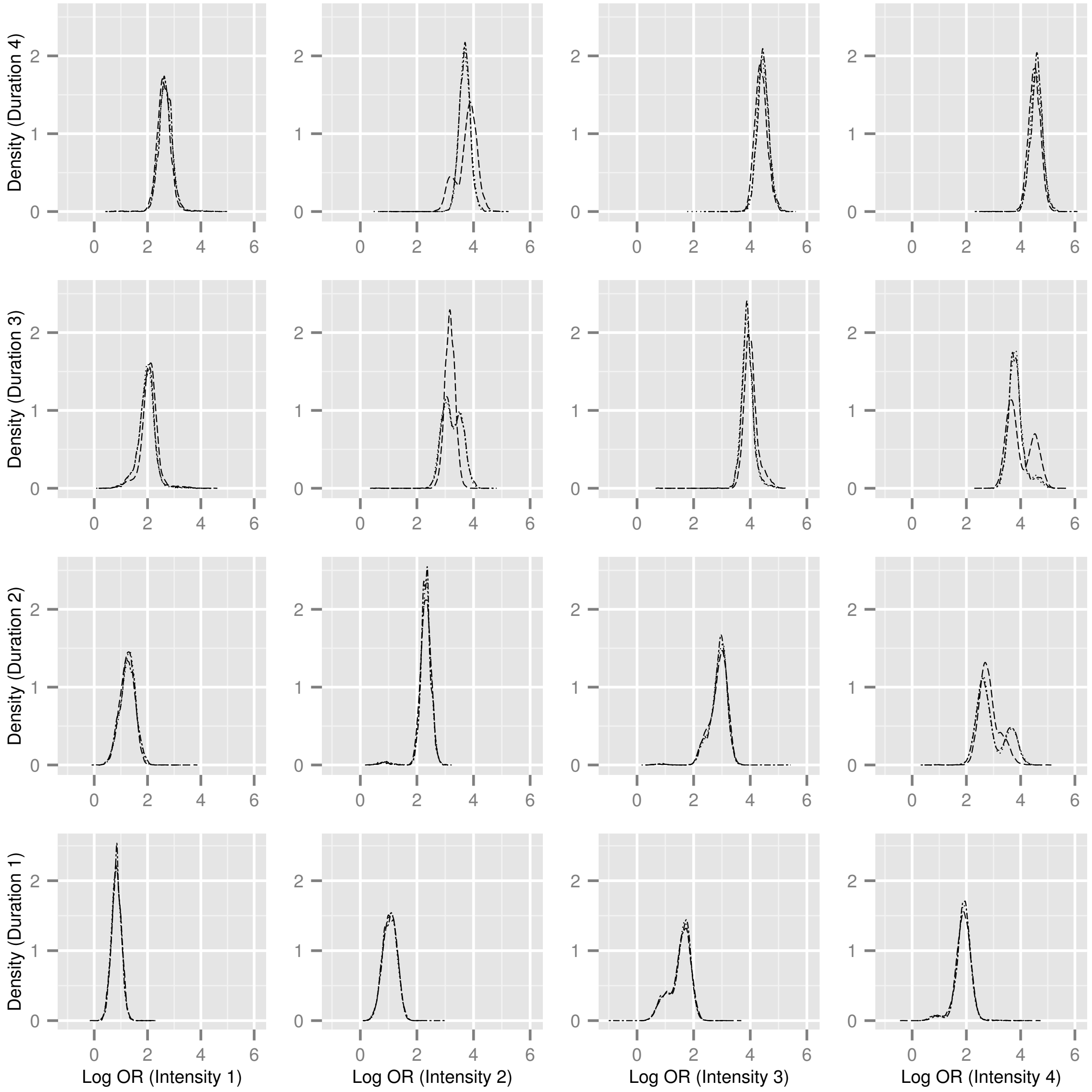

Figure 5: Log odds ratio relative to the non-smoking cluster 1, for the clusters in the representative clustering of the analysis of the alternative discretisation of intensity, duration, time since cessation and pack years.

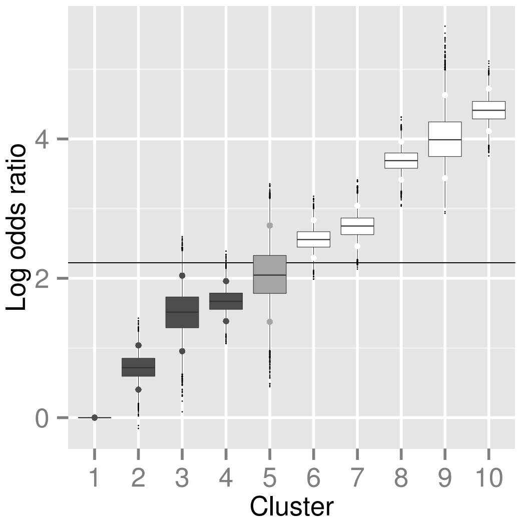

Figure 6: Density estimates of predicted log odds ratios relative to a non-smoking profile, for different intensity and duration combinations discretised differently. Time since cessation and pack years treated as missing in pseudo-profiles.

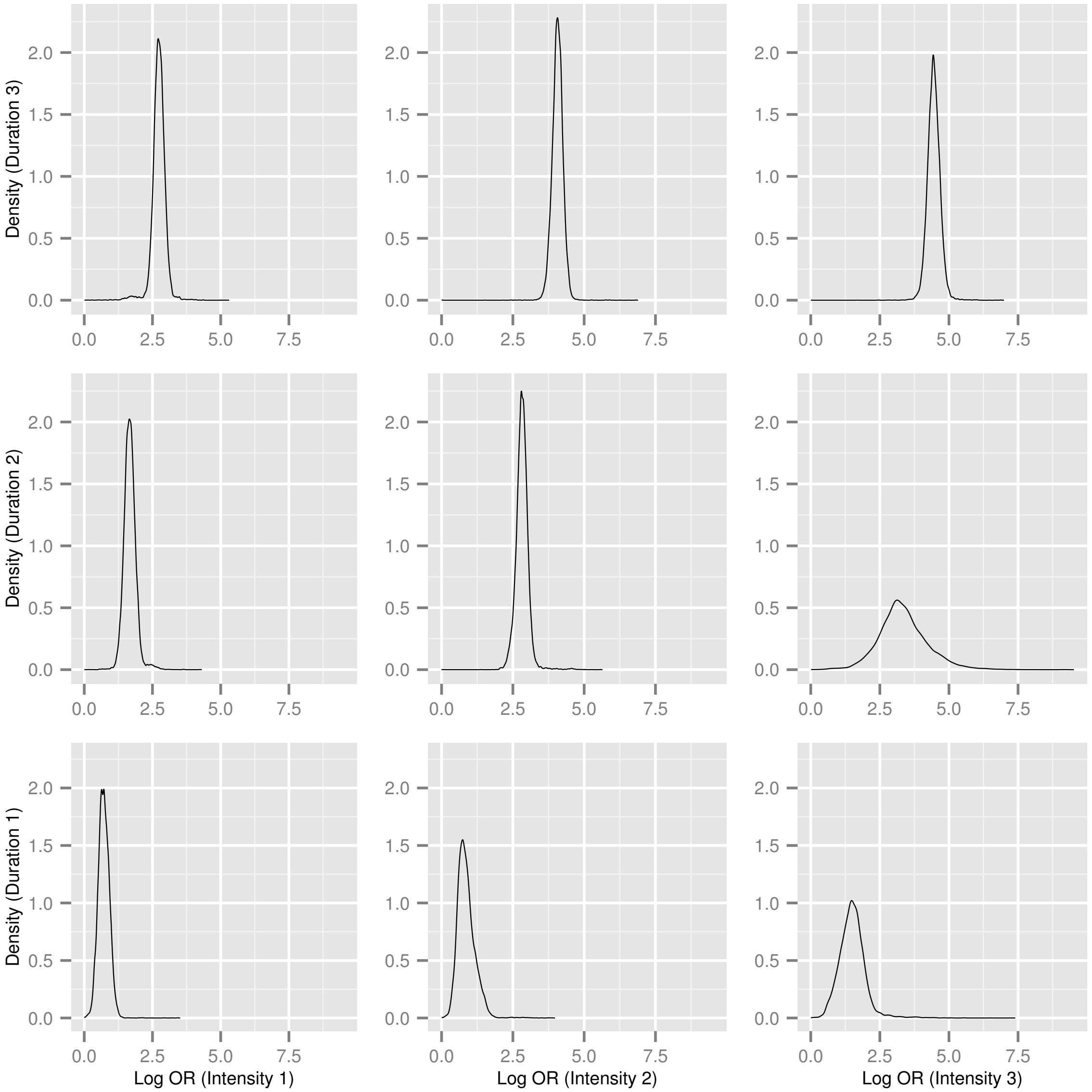

Figure 7: Plot of indivduals against their first and second principal component values. Individuals are coloured by which cluster in the representative clustering they belong to.

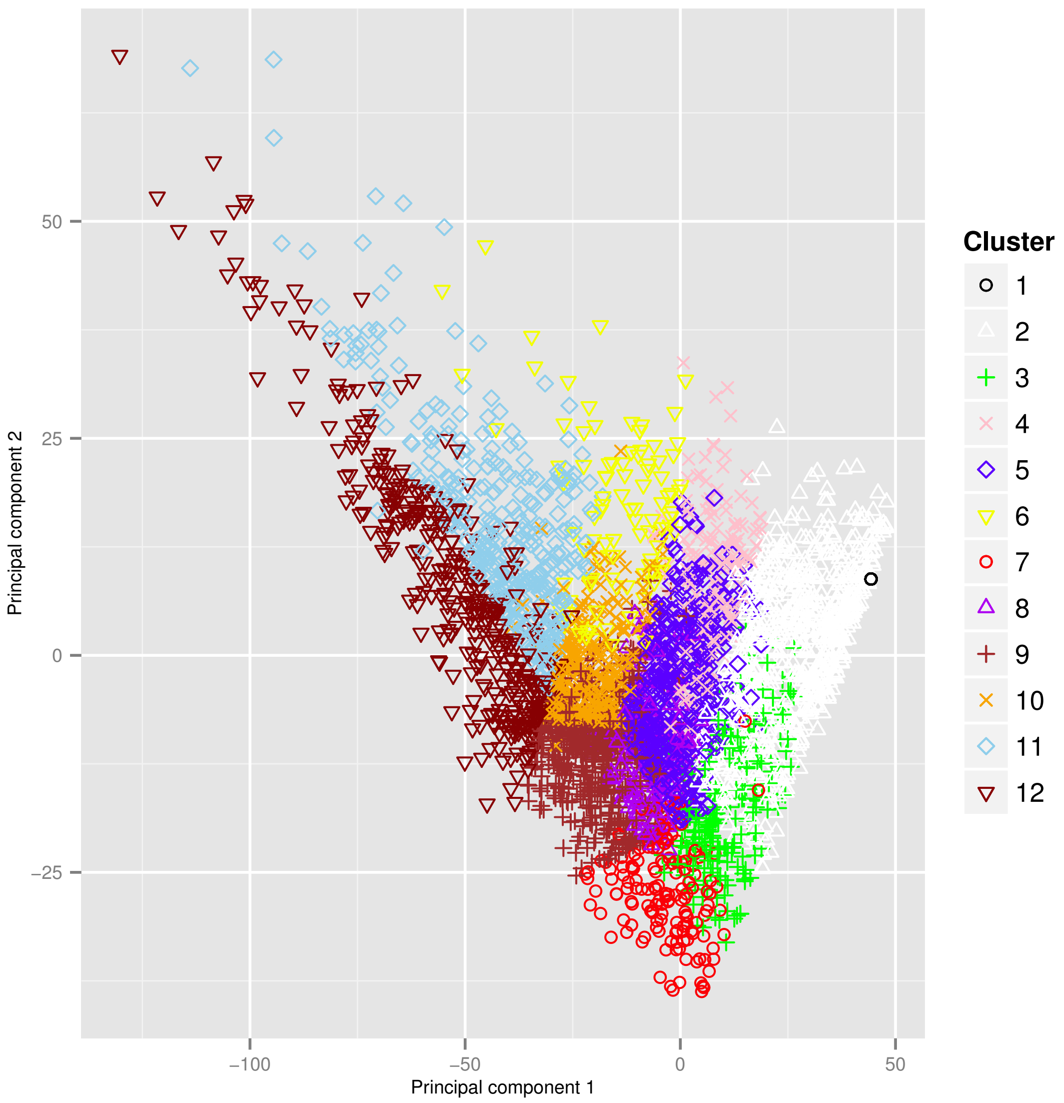

eTables

Covariate	Category id	Category Description	N.Subjects
Average intensity of smoking	0	Non-smoker	823
	1	$0 <$ cigarettes per day ≤ 15	1307
	2	$15 <$ cigarettes per day ≤ 30	1963
	3	$30 <$ cigarettes per day	550
	NA	Not available	15
Duration of smoking	0	Non-smoker	823
	1	$0 <$ years ≤ 15	642
	2	$15 <$ years ≤ 35	1728
	3	$35 <$ years	1465
Time since quit smoking	0	Non-smoker	823
	1	$15 <$ years	1156
	2	$5 <$ years ≤ 15	661
	3	$0 <$ years ≤ 5	632
	4	Current smoker	1386
Pack-years	0	Non-smoker	823
	1	$0 <$ pack-years ≤ 15	1813
	2	$15 <$ pack-years ≤ 30	589
	3	$30 <$ pack-years ≤ 45	781
	4	$45 <$ pack-years	633
	NA	Not available	19

Table 1: Summary of covariate categories. The categories used in the alternative discretisation for applying profile regression to data from the ICARE case-control study

Table 2: Summary of cluster profiles. Table of distribution means for characteristics of clusters from the representative clustering of the analysis of the alternative discretisation of intensity, duration, time since cessation and pack-years. For the covariates, the distribution is of the probability that the covariate is in each category.

		Cluster									
		1	2	3	4	5	6	7	8	9	10
No. Subjects		823	582	63	543	105	699	390	692	137	624
Log OR		0	0.72	1.50	1.67	2.05	2.55	2.74	3.68	4.00	4.41
INT	0	1.00	0.00	0.01	0.00	0.00	0.00	0.00	0.00	0.00	0.00
	1	0.00	0.65	0.02	0.92	0.01	0.04	0.97	0.01	0.00	0.00
	2	0.00	0.32	0.55	0.07	0.12	0.93	0.02	0.97	0.07	0.54
	3	0.00	0.01	0.40	0.00	0.85	0.02	0.00	0.00	0.91	0.44
DUR	0	1.00	0.00	0.01	0.00	0.00	0.00	0.00	0.00	0.00	0.00
	1	0.00	0.95	0.84	0.03	0.02	0.01	0.00	0.00	0.00	0.00
	2	0.00	0.04	0.12	0.86	0.92	0.95	0.12	0.36	0.88	0.05
	3	0.00	0.00	0.01	0.09	0.03	0.03	0.87	0.63	0.10	0.94
TSC	0	1.00	0.00	0.01	0.00	0.00	0.01	0.01	0.00	0.01	0.00
	1	0.00	0.82	0.78	0.39	0.42	0.37	0.03	0.06	0.17	0.02
	2	0.00	0.09	0.13	0.19	0.33	0.21	0.16	0.17	0.24	0.14
	3	0.00	0.02	0.03	0.11	0.09	0.11	0.25	0.26	0.15	0.26
	4	0.00	0.05	0.03	0.29	0.14	0.29	0.54	0.49	0.41	0.56
PY	0	1.00	0.00	0.01	0.00	0.00	0.01	0.01	0.00	0.00	0.00
	1	0.00	0.98	0.07	0.73	0.01	0.01	0.26	0.00	0.00	0.00
	2	0.00	0.01	0.85	0.24	0.07	0.78	0.59	0.07	0.01	0.00
	3	0.00	0.00	0.03	0.01	0.41	0.17	0.13	0.88	0.16	0.04
	4	0.00	0.00	0.02	0.00	0.49	0.01	0.00	0.04	0.80	0.94

Table 3: Comparison of methods. Comparison of measures of fit for profile regression, logistic regression and CART for our case-control simulation study.

**Scenario 1**					
	RMSEp	MAEp	RMSEy	MAEy	Misclassification error
profile regression	0.14	0.10	0.47	0.38	0.30
logistic regression	0.14	0.10	0.48	0.46	0.39
CART	0.17	0.14	0.49	0.48	0.41
**Scenario 2**					
	RMSEp	MAEp	RMSEy	MAEy	Misclassification error
profile regression	0.01	0.01	0.47	0.44	0.35
logistic regression	0.12	0.11	0.48	0.47	0.41
CART	0.01	0.01	0.47	0.44	0.38

eReferences

e1. Hastie DI, Liverani S, Richardson S. `PReMiuM`: Dirichlet Process Bayesian Clustering, Profile Regression. 2013. `R` package version 3.0.20.

e2. R Core Team. `R`: A language and environment for statistical computing. R Foundation for Statistical Computing, Vienna, Austria. 2012. ISBN 3-900051-07-0, URL http://www.R-project.org/.

e3. Hastie DI, Liverani S, Richardson S. Sampling from Dirichlet process mixture models with unknown concentration parameter. 2013. Submitted.

e4. Ripley B. `tree`: Classification and regression trees. 2012. `R` package version 1.0-33.
